# Attitudes of people diagnosed with cancer and cancer care providers towards use of nicotine vaping products in high-income countries: a scoping review

**DOI:** 10.1007/s11764-024-01601-7

**Published:** 2024-04-17

**Authors:** Lavender A. Otieno, Jeffin Baiju, Joshua Trigg

**Affiliations:** College of Medicine and Public Health, Flinders Health and Medical Research Institute, Flinders University, Bedford Park, South Australia Australia

**Keywords:** Nicotine vaping, Cancer, Oncology, Risk attitudes

## Abstract

**Purpose:**

To investigate the attitudes, beliefs and perceptions of people diagnosed with cancer and health practitioners on use of nicotine vaping products.

**Methods:**

Scopus and OVID Medline were searched for papers published between 2013 and 2023. Two authors independently selected the studies and extracted data, with conflicts resolved through discussion. Nine studies were selected for further synthesis. Reporting follows the PRISMA Scoping Reviews checklist.

**Results:**

E-cigarettes were commonly perceived as less harmful compared to conventional cigarettes and less detrimental to cancer treatment effectiveness among people with a current or previous cancer diagnosis. This population also cited smoking cessation, smoking in non-smoking areas and less risky alternative as the most common reasons for e-cigarette use. Nevertheless, low levels of clinician support on the effectiveness of e-cigarettes as a smoking cessation tool/alternative were identified.

**Conclusion:**

Findings show differences in beliefs and attitudes of e-cigarettes between clinicians and people diagnosed with cancer. Additional research into the health impacts of e-cigarettes in people with a current or previous cancer diagnosis will allow for greater congruence between patients and clinicians and assist providers in recommending effective tools for smoking cessation within this population.

**Implications for Cancer Survivors:**

This study provides an overview of the attitudes, beliefs and perceptions of e-cigarette use among people with a current or previous diagnosis of cancer and health practitioners. Given the increased prevalence of e-cigarette use within this population, these findings highlight a greater need for dialogue between patients and clinicians regarding the safety and efficacy of these devices.

## Introduction

Tobacco smoking is a known risk factor for several cancers including lung, pancreatic, bladder, cervical, stomach and gastrointestinal cancers [1–3]. Continued smoking following a cancer diagnosis is one of the strongest predictors of survival time and is associated with detrimental outcomes [4]. These outcomes include increased likelihood of recurrence, development of secondary primary cancers, reduced treatment efficacy and death [5–8]. Despite this, approximately 33% of patients continue to smoke after a cancer diagnosis, or relapse to smoking after attempting to quit [4, 9].

The overall global trend of e-cigarette use has shown an upward trend from 2011 to 2019 with current global lifetime and current prevalence of e-cigarette use at 23% and 11% respectively [10]. In Australia, about 11% of the general population aged 14 and over reported ever use of e-cigarettes in 2019 [11]. Ever use was highest among young adults aged 18 to 24 years old at 26%, with lower use among older people [11]. Pooled and weighted data from Australia’s five major cities (Sydney, Melbourne, Brisbane, Adelaide and Perth) also showed a gradual increase in current use of e-cigarettes between 2020 and 2023 (2 to 8.9%) in population aged 14 years and over [12]. Among people previously diagnosed with cancer, aged 18 to 44 years old, US studies showed that the prevalence of e-cigarette use is estimated at 12% [13]. Furthermore, the US National Health Interview survey (2014–2018) found that 23% of people previously diagnosed with cancer actively used e-cigarettes [14]. A recent systematic review and meta-analysis showed that approximately 15% of people previously diagnosed with cancer between 2005 and 2019 reported lifetime use of e-cigarettes, with a higher prevalence in younger people aged < 45 years (46.7%) compared to older people aged ≥ 65 years (24.8%) [15]. Additionally, one study found that 63% of people previously diagnosed with cancer who currently use conventional cigarettes also used e-cigarettes, suggesting dual use in this population [15]. This rise may reflect lower perceived health risks of vaping as a tobacco smoking cessation approach that gradually replaces conventional cigarettes with e-cigarettes.

Nicotine vaping products (NVPs) are a source of known toxic components such as heavy metals, metalloids, nitrosamines and aldehydes [16–18]. In vitro and in vivo studies show associations between respiratory conditions such as asthma, increased inflammatory response, oxidative and DNA damage and the aerosol produced during vaping [19]. Although tobacco smoking has higher risk profile for these domains, the health risks associated with NVPs cannot be ignored. Given the increased risk of developing health conditions such as cardiovascular and pulmonary diseases among people diagnosed with cancer during treatment [20], e-cigarette use may prove problematic given that the composition of these products has the potential to affect the development, course and prognosis of these health issues in people diagnosed with cancer. It is therefore imperative to understand the perceptions, attitudes and beliefs of people diagnosed with cancer towards the use of NVPs. We conducted a scoping literature review using a systematic search strategy to investigate the attitudes, beliefs and perceptions of NVPs among people diagnosed with cancer and their cancer care providers, and summarise key themes addressed in this literature. A secondary aim was to note any data on findings relative to information sources used regarding NVPs and prevalence of NVPs use.

## Methods

### Search strategy

Search terms were developed by reviewing published works for terms involving cancer, NVPs, attitudes, beliefs, or perceptions of NVP use. Research librarians were consulted to develop and refine the search strategy, which is provided in Table 1. Search terms related to “nicotine vaping products”, “perception”, “cancer” and “cancer patients and support” were selected. We identified relevant publications by searching the Scopus and OVID Medline databases for articles published from 2013 to 2023, with final searches run on 22 September 2023.

**Table 1:** Search terms by construct

Core concepts	Search strings
Nicotine vaping products	"electronic nicotine delivery systems" or "vaping" or "e-cigs" or "e-cigarette*" or vape* or vapor or "e-hookahs" or "vape pens" or "electronic cigarettes" or "heat-not-burn" or iqos or nicotine or "alternative nicotine products" or "nicotine vaping products" or "electronic nicotine delivery devices" or "electronic non-nicotine delivery device"
Perception	attitud* or belie* or view* or perspective or valu* or perce* or impact or reflection or experience or implication or influence or effect or awareness
Cancer	neoplasms or cancer* or melanoma* or myeloma* or sarcoma* or lymphoma* or neuroblastoma* or retinoblastoma* or osteosarcoma* or tumor* or tumour* or malignan* or neoplas* or leukemi* or leukaemi* or carcinoma* or adenocarcinoma*
People diagnosed with cancer and support	patients or survivors or clients or doctor* or physician or practitioner or nurs* or caregiver or family or relative

### Inclusion and exclusion criteria

Articles were required to be available in full text, published in English and address perceptions, attitudes, or beliefs relating to NVP use among people with a current or previous cancer diagnosis or clinicians. No specification of cancer type or age was required. Studies from Australia, the USA, UK, Canada and New Zealand were included, to meet the high-income country focus, with other countries excluded. Primary studies using qualitative and quantitative methodologies were included, provided they reported subjective views (i.e., beliefs, attitudes, or perceptions) about NVP use among people with a current or previous diagnosis of cancer. Reviews, preprints, commentaries, conference abstracts, dissertations and grey literature were not included. Further eligibility criteria and participant information are presented in Tables 2 and 3.

**Table 2:** Characteristics of included studies

Author, year	Study design	Country/setting	Sample size	Recruitment period	Primary outcome	Secondary outcomes	Data source	Funding/Interests
Bjurlin, 2022 [22]	Cross-sectional survey	USA	11,847 respondents (26.6% reported cancer diagnosis)	2017–2019	E-cigarette harm perception relative to conventional cigarettes	NR	Health Information National Trends Survey (HINTS)	Lineberger Comprehensive Cancer Centre Innovation award, and a NC TraCS grant The authors declare no conflict of interest
Brett, 2020 [23]	Cross-sectional survey	UK	506 clinicians	2018–2019	E-cigarette knowledge and beliefs	Recommendation of e-cigarettes	All clinicians registered with doctors.net.uk	Cancer research UK Ref: A24355 NIHR Oxford Biomedical Research Centre and the Applied Research Centre The authors declare no conflict of interest
Buczek, 2018 [24]	Cross-sectional survey	USA	979 people who currently smoke (39 people diagnosed with cancer)	2012–2013	Perception of e-cigarettes relative to conventional cigarettes	E-cigarette advertising and information exposure	National Cancer Institute-designated Comprehensive Cancer centre-associated hospital	National institute on Drug Abuse The authors declare no conflict of interest
Correa, 2018 [25]	Cross-sectional survey	USA	121 people diagnosed with cancer	2016–2017	E-cigarette patterns and beliefs	Communication with oncology providers	H. Lee Moffitt Cancer Center	National Cancer Institute, Grant/Award Numbers: R01 CA154596 and P30CA76292 Thomas H. Brandon received research support from Pfizer, Inc. All other coauthors declare no conflict of interest
Fucito, 2021 [26]	Cross-sectional survey	USA	150 people diagnosed with cancer	2019	E-cigarette perceptions and interests	NR	Cancer hospital and affiliated ambulatory care centre	National Institutes of Health grants, National Institutes of Health and FDA Centre for Tobacco products The authors declare no conflict of interest
Jackson, 2020 [27]	Cross-sectional survey	USA	847 people with a previous cancer diagnosis	2019	E-cigarette beliefs and perceptions	Nicotine and low nicotine cigarette beliefs	Health Information National Trends Survey (HINTS)	Komen Graduate Training and Disparities Research Program The authors declare no conflict of interest
Kalkhoran, 2018 [28]	Cross-sectional survey	USA	302 people diagnosed with cancer	2013–2017	E-cigarette reasons for use	Prevalence, correlates and frequency of e-cigarette use	Massachusetts General Hospital, Dana Farber Cancer Institute and Memorial Sloan Kettering Cancer Center, New York	CVS Foundation, Drs. Kalkhoran, Rigotti, Ostroff and Park receive royalties from UpToDate. Dr Ostroff also receives research support from the CVS Foundation. Dr Kruse has a financial interest in Dimagi, Inc and is a paid consultant for Click Therapeutics
Kalkhoran, 2022 [29]	Longitudinal mixed methods design RCT	USA	303 people diagnosed with cancer	2013–2017	Smoking cessation	Association between smoking cessation and e-cigarette use	Two NCI-designated Comprehensive Cancer Centres	National Cancer Institute and National Heart, Lung and Blood Institute
Sherratt, 2016 [30]	Cross- sectional survey	UK	147 clinicians	2015	E-cigarette attitudes and perception	E-cigarette use and recommendation	British Thoracic Oncology Group (BTOG) website	Liverpool Clinical Commissioning Group

**Table 3:** Characteristics of participants in included studies

Study	Population	Sample size	Age (mean/range)	Ethnicity (% or *n*)	Gender (% or *n*)	Nicotine use
Bjurlin, 2022 [22]	Adults in the civilian, non-institutionalised population of the USA. Cancer survivors was defined as individuals who confirmed ever being diagnosed as having cancer	11,847 respondents (26.6% reported cancer diagnosis)	Overall range: 18 years or older (*n*): Aged 18–34 years = 1443 Aged 35–49 years = 2266 Aged 50–64 years = 3830 Aged 65–74 = 2578 Aged 75 or older = 1673	White (*n*) = 7188 Black (*n*) = 1766 Other (*n*) = 2545	Female (*n*) = 6429 Male (*n*) = 4621 Nonbinary (NR)	Cigarette use (*n*): Current = 1457 Former = 3054 Never = 7177 E-cigarette use (*n*): Current = 338 Former = 1127 Never = 10,190
Brett, 2020 [23]	Clinicians (GPs, oncologists, cancer surgeons, practice nurses and cancer nurse specialists)	506	NR	NR	Female = 57.5% Male = 41.1% Nonbinary (NR)	Tobacco use (%): Current = 2% Former = 21.3% Never = 72.3% Current e-cigarette use (%) = 1.2%
Buczek, 2018 [24]	Adults with a verified current smoking status and a documented cancer diagnosis based on ICD-9 codes	979 (39 people diagnosed with cancer)	Overall mean = 49.3 years	White = 56.4% Black = 38.5% American Indian = 2.6% Native Indian = 2.6%	Female = 59% Male = 41% Nonbinary (NR)	E-cigarette use (%): Ever use = 46.2% Current use = 38.9%
Correa, 2018 [25]	Adults with a cancer diagnosis of any type (except basal cell carcinoma) that is being actively treated	121	Overall mean (SD) = 55.6 (9.9) years	White = 88.4%	Female = 56.2% Male = 43.8% Nonbinary (NR)	Participants smoked ≥ 100 lifetime cigarettes and used an e-cigarette in the past 30 days (current users) Dual use = 51%
Fucito,2021 [26]	Individuals attending an initial lung cancer screening appointment	150	Overall mean (SD) = 61.8 (4.8) years	Non-Hispanic/non-Latino/non-Latina = 87.9% Hispanic/Latino/Latina = 9.3% White = 67.3% Black = 25.2%	Female = 40.2% Male = 59.8% Nonbinary (NR)	Tobacco use: Participants reported current smoking (i.e. ≥ 1 combustible cigarettes within past 30 days) E-cigarette ever use = 28%
Jackson, 2020 [27]	Adults in the civilian, non-institutionalised population of the USA. Cancer survivors was defined as individuals who confirmed ever being diagnosed as having cancer	847	Overall range: 18 years or older (*n*): Aged 18–34 years = 329 Aged 35–49 years = 500 Aged 50–64 years = 889 Aged 65 or over = 995	Non-Hispanic white (*n*) = 1723 Non-Hispanic black (*n*) = 280 Hispanic (*n*) = 309 Non-Hispanic other (*n*) = 178	Female (*n*) = 1276 Male (*n*) = 1243 Nonbinary (NR)	Ever used cigarettes (*n*) = 1868 Ever used e-cigarettes (*n*) = 651
Kalkhoran, 2018 [28]	Adults with a recent diagnosis of cancer (within 3 months of diagnosis or four visits to the cancer centre)	302	All: NR Current e-cigarette use (Mean (SD)) = 56.8 (10.2) years: Former e-cigarette use (Mean (SD)) = 57.7 (10.1) years Never e-cigarette use (Mean (SD)) = 59.2 (9.3) years	White (*n*) = 254 Black (*n*) = 30 Other (*n*) = 18 Hispanic (*n*) = 12	Female (*n*) = 169 Male (*n*) = 133 Nonbinary (NR)	Participants smoked at least one cigarette in the past 30 days (current users) E-cigarette use (*n*): Current = 56 Former = 93 Never = 153
Kalkhoran, 2022 [29]	Adults with a recent diagnosis of cancer (within 3 months of diagnosis or four visits to the cancer centre)	303	Median: 39 years	Non-Hispanic white = 84%	Female = 56% Male = 44% Nonbinary (NR)	Participants smoked at least one cigarette in the past 30 days (current users) Baseline current e-cigarette use = 19%
Sherratt, 2016 [30]	Practitioner members of the BTOG (Medical and clinical oncologists, respiratory physicians, surgeons, radiotherapists, radiologists, nurses, pharmacists and scientists)	147	Overall range = 18 years or older: *n* (%) Aged 18–29 years = 5 (3.4); Aged 30–49 years = 89 (60.5); Aged 50–69 years = 50 (34); Aged ≥ 70 years = 3 (2)	NR	Female = 64.6% Male = 35.4% Nonbinary (NR)	Tobacco use *n* (%): Ever = 44 (29.9) Never = 103 (70.1) E-cigarette use *n* (%): Ever = 14 (9.6) Never = 132 (90.4)

### Study selection

**Fig. 1:**
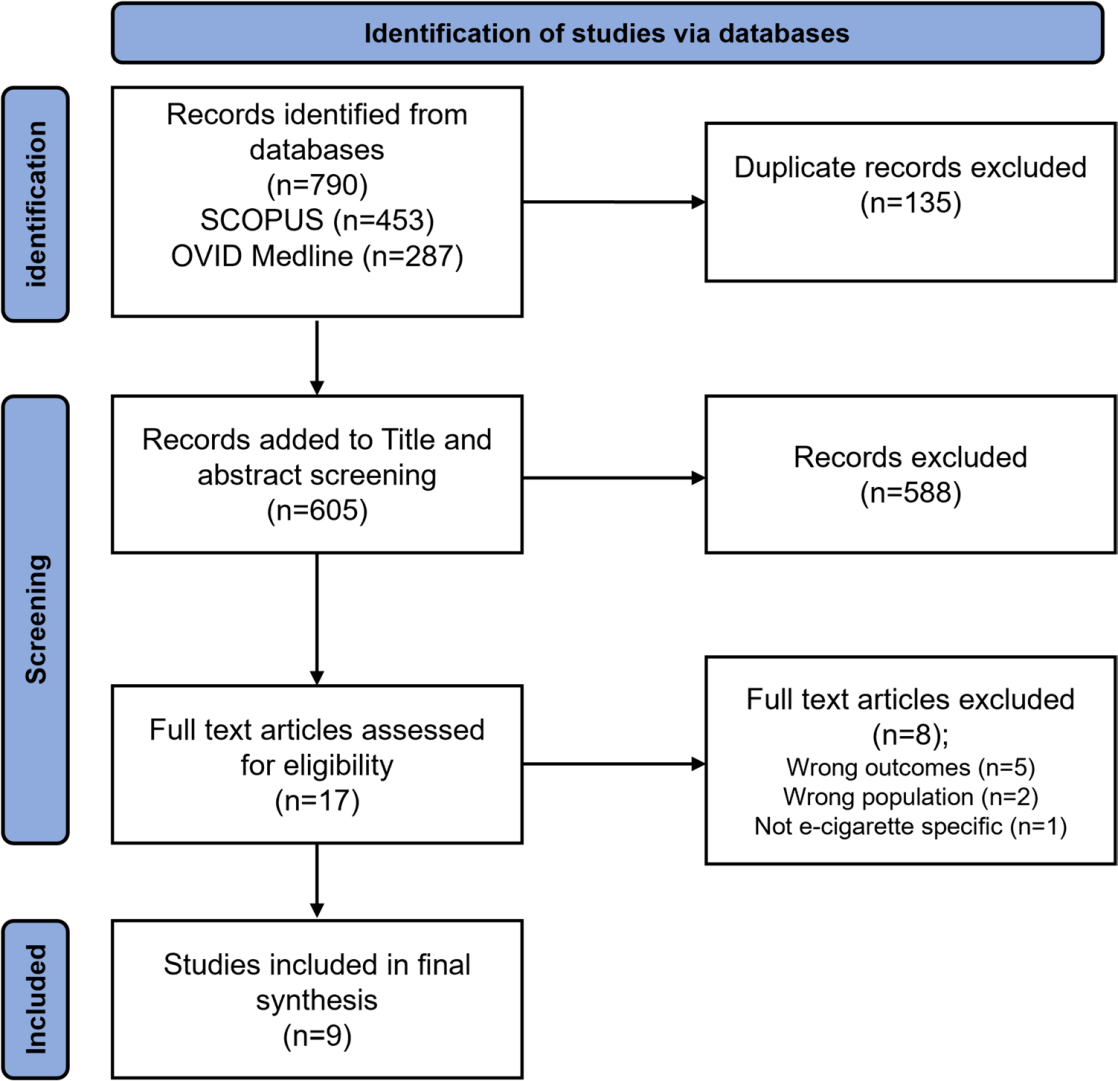
Diagram of study identification, screening and selection

Figure 1 displays the flow chart for selection. The search and selection of articles followed PRISMA-ScR guidelines for scoping reviews [21]. The initial set of articles were deduplicated in Covidence, with the final set of unique papers undergoing title, abstract and full-text screening against inclusion and exclusion criteria in Covidence. All literature was reviewed by JB and cross-checked by JT against inclusion and exclusion criteria, with discrepancies resolved by group discussion (LO, JB, JT).

### Data extraction

A data extraction tool was created in Covidence to summarise study characteristics, including publication year, country of origin, participant characteristics, study design, aims and main findings for all studies that included a quantitative or qualitative component. Extraction was completed independently by two researchers (JB, LO) and any discrepancies resolved by group discussion (JT, LO, JB). Findings were extracted to a descriptive summary table (Tables 2 and 3).

### Data analysis

Findings from all included studies were narratively synthesised, given the range of methodologies and populations addressed in the final set of studies, and to identify key concepts across the studies. Study outcomes were first categorised according to initial themes, and these were discussed among the study team. We then refined and named the final themes, with one author (LO) summarizing included studies in a narrative framework. This framework included the data extraction fields and factors relating to risk perception, smoking cessation, social interaction and NVP use context. This framework was then discussed in relation to the original themes for consensus.

## Results

Nine articles met the eligibility criteria for inclusion in this review, following screening of 790 studies, with 605 unique studies screened by title and abstract, and 17 unique studies screened by the full text (Table 2). Studies were excluded at full text due to being out of scope, or by originating country and population. The included studies spanned from 2018 to 2022, with seven studies from the USA and two from the UK. Other high-income countries were not represented in studies eligible for inclusion. Eight quantitative and one mixed methods study with a qualitative component were identified. No solely qualitative studies were eligible for inclusion. Two studies assessed clinician perspectives, while seven studies assessed perceptions of people with a current or previous cancer diagnosis. Beliefs assessed included relative harm in comparison to cigarettes, smoking cessation aid and use during cancer treatments. The aim of this study was to scope the literature; therefore, study quality was not assessed.

### Perception of NVPs

Four studies examined perceptions or attitudes towards e-cigarettes among people with a current or previous cancer diagnosis [22, 24, 25, 27]. Of these studies, three reported reduced harm perception and risk belief of e-cigarettes compared to conventional cigarettes [24, 25, 27], and one study reported a greater harm perception of e-cigarettes compared to conventional cigarettes among people diagnosed with cancer [22]. One study showed that current users considered e-cigarettes to be less detrimental to their cancer treatment effectiveness and less likely to increase the risk of cancer treatment-related problems, relative to smoking [25]. One study also noted that participants (with and without a cancer diagnosis) who believed that e-cigarettes were just as or more harmful than conventional cigarettes were less likely to ever use e-cigarettes compared to those who believed that they were relatively less harmful [27]. Among those with a history of cancer, the belief that e-cigarettes were more harmful than conventional cigarettes was associated with lower likelihood of having ever used e-cigarettes (aOR 0.16; 95% CI; 0.04–0.57) [27]. Four studies assessed possible reasons for e-cigarette use among people diagnosed with cancer [24, 25, 28, 29]. Smoking cessation or reduction was the most common reason given for e-cigarette initiation and continued use, followed by ability to use nicotine in non-smoking areas, and use of a perceived lower risk alternative to tobacco. One study reported that 21% of people diagnosed with cancer initiated use due to health concerns related to their cancer diagnosis [25].

### Clinician attitudes towards NVPs as smoking cessation tools

Two studies also assessed clinician attitudes and beliefs towards e-cigarettes [30, 31]. One study (*n* = 147) [30] reported that practitioners were most likely to advise that e-cigarettes were less harmful than regular cigarettes (23.7%). Practitioners also reported a scarcity in research and uncertainty regarding possible health effects associated with e-cigarette use (21.6%). Some practitioners provided no advice to patients or suggested that they had inadequate knowledge to advise patients (6.3%), while 3.7% stated that they would encourage use and 5.8% would discourage use [30]. The second study [31] reported that 29% of clinicians (*n* = 506) would not recommend e-cigarettes to patients with cancer who smoke tobacco, 51% would recommend e-cigarettes as an interim option to patients, while 20% would recommend e-cigarettes as a partial replacement for smoking [31]. Many clinicians (42%) felt uncomfortable discussing e-cigarettes with their patients, and 38% noted that most of their colleagues would also feel uncomfortable recommending e-cigarettes to patients with a cancer diagnosis. Furthermore, 37% were unsure whether clinicians should discourage patients with a cancer diagnosis from using e-cigarettes [31].

Many clinicians and healthcare professionals reported low levels of confidence or insufficient knowledge regarding e-cigarette related advice, with the vast majority stating that they need more information and guidance regarding e-cigarette advice to patients [30, 31]. Uncertainty regarding the safety of e-cigarettes in comparison to conventional cigarettes or tobacco was also evident in both studies [30, 31]. One study reported inconsistent advice given to patients by practitioners [30]. Almost one in four (24%) of practitioners (*n* = 141) advised patients that e-cigarettes were less likely to be harmful compared to conventional cigarettes despite insufficient evidence available to inform the adverse effects of e-cigarette use. On the other hand, 6% of clinicians provided no advice [30].

While participants in one study stated that they felt comfortable discussing e-cigarettes with their oncology provider and believed it was important to do so, most participants did not know their provider’s stance because it was never discussed [25]. Less than half (*n* = 121) told their providers about their e-cigarette use, and only 24% reported being asked about their usage [25]. Participants in a separate study reported speaking to their physician about e-cigarette use and noted that their doctors do not support use due to perceptions of harm or lack of knowledge about harms and safety [29]. Some participants also perceived that their doctor viewed e-cigarettes as more harmful than conventional cigarettes [29].

### Information sources

In one study, all people diagnosed with cancer (*n* = 39) had heard of e-cigarettes and reported initial exposure from various sources [24]. Common sources of e-cigarette information included television (77%), stores (49%), friends (36%), family (31%), internet advertisements (10%), magazines (5%), radio (5%), newspaper (5%) and healthcare providers (5%) [24].

Regarding health practitioners, many clinicians in one study (*n* = 506) had sought information about e-cigarettes from government or health agencies (55%), professional associations (37%), healthcare colleagues (29%), news media or advertising (24%), in scientific literature (23%), or professional development training (22%) and charities (18%). Notably, 19% of clinicians reported having never sought information about e-cigarettes [31].

### Prevalence of NVP use among people with a current or previous cancer diagnosis

All studies identified prevalence of e-cigarette use among people with a current or previous cancer diagnosis [22, 24–31]. Two studies compared nature and frequency of e-cigarette use between people with a previous cancer diagnosis and those without a cancer history [22, 27] with one large study (*n* = 11,847) reporting current e-cigarette use in 2.9% (95% CI 1.4–4.3) of people with a previous cancer diagnosis and 5% (95% CI 4–6) in respondents without a cancer diagnosis [22]. However, similar overall distinctions in frequency and nature of use were not clearly defined in the second study [27]. Other studies focused solely on people with a current or previous diagnosis of cancer [24–26, 28–31] with up to 49% of participants (*n* = 302) reporting ever use and 19% reporting current use [28]. In two studies, up to 42% of practitioners and clinicians estimated regular use of e-cigarettes among 25% or more of the ever-smoking patients seen in the past year [30, 31]. One study also reported dual use of tobacco cigarettes and e-cigarettes among 51% of people diagnosed with cancer (*n* = 121 current users) [25].

## Discussion

This scoping review sought to examine attitudes, beliefs and perceptions of people diagnosed with cancer and their health practitioners on NVP use. Findings revealed lower harm perception of e-cigarettes compared to conventional cigarettes among people with a current or previous diagnosis of cancer. Reasons for use included smoking cessation, nicotine use in non-smoking areas and lower perceived health risk relative to conventional cigarettes. Findings also suggested that most clinicians would not recommend e-cigarettes to their patients due to low confidence in the efficacy and safety of e-cigarettes as well as insufficient knowledge. Prevalence of e-cigarette use remains quite high among people with a current or previous cancer diagnosis, with dual use reported in just one study.

Studies show an association between reduced harm perception of e-cigarettes and greater odds of e-cigarette initiation or use among young people [32–35]. It is likely that the reported reduced harm perception of e-cigarettes may influence prevalence of use of these products among people with a current or previous cancer diagnosis, particularly among people who currently smoke or have smoked in the past. Nevertheless, reasons frequently given for e-cigarette use among patients include smoking cessation and use of a perceived lower risk alternative. Continued smoking during cancer treatment is associated with detrimental outcomes [4], such as cancer recurrence, secondary primary cancers and reduced treatment efficacy [5–8]. Considering e-cigarettes as less detrimental to treatment effectiveness and less likely to increase risk of treatment complications [25] may increase the likelihood of using e-cigarettes in this context. Although evidence exists to support the use of e-cigarettes as a smoking cessation tool [36, 37], the application of this for people with a cancer diagnosis who aim to quit smoking requires more evidence, particularly for longer-term outcomes. Interestingly, the only longitudinal smoking cessation trial selected for synthesis in this review showed that e-cigarette use during trial participation was not associated with smoking abstinence at 6 months among individuals who smoke and have been recently diagnosed with cancer [29].

Clinicians and healthcare workers also expressed apprehension regarding NVPs. Findings suggested that clinicians remained uncertain about the safety and efficacy of e-cigarettes and viewed these products as just as harmful or more harmful than conventional cigarettes. This sentiment is echoed in different medical fields [13, 38, 39]. However, unlike current findings, we do see an increased likelihood of physicians such as general practitioners recommending e-cigarettes to people who smoke in other patient groups depending on age, smoking status and prior use of nicotine replacement therapy [13, 40] despite sharing similar uncertainties about the efficacy of e-cigarettes. The increased risk of e-cigarette use on health or treatment outcomes of people diagnosed with cancer compared to other patient groups is likely to inform hesitance to suggest NVPs for smoking cessation.

Differences in harm perception and attitudes were identified between patients and clinicians in this review, which may be attributed to information sourcing. While patients obtain information from television, stores, friends and family [24], clinicians tend to rely on health-related sources such as scientific literature, government or health agencies and professional colleagues [31]. Despite accessing scientific literature, clinicians still expressed a lack of confidence in discussing e-cigarette related information with patients. One study observed an association between a greater frequency of discussing patients’ e-cigarette use and greater perceived knowledge of e-cigarettes among health practitioners [41]. The same study also showed that nursing practitioners and physician assistants were more likely to discuss e-cigarette use with patients compared to oncologists [41]. Currently, clinical guidelines on smoking cessation are being adapted to incorporate evidence and practice considerations for patients’ use of NVPs to quit smoking [42]; however, there is a need for further evidence to inform use among people currently or previously diagnosed with cancer to facilitate discussions between health practitioners and patients. The development of accessible and reliable health materials and campaigns is also required to bridge the difference in harm perceptions of NVPs between people diagnosed with cancer and their healthcare providers.

This scoping review is not without limitations. Studies captured in this review were not able to be directly synthesised (e.g. across age groups), due to methodological variation and use of different outcome measures. Studies in this review also mainly evaluated knowledge, attitudes or perception of e-cigarettes but not the impact of perception on initiation or frequency of use in people diagnosed with cancer. This was largely due to the deliberate focus on subjective and attitude outcomes, rather than standardised outcome measures. As all but two studies were from outside the USA, the findings have less relevance to lower income countries, and further research on this is warranted. One other important consideration is the prevalence of NVP use among clinicians. Although this lies beyond the scope of this review, it is an important consideration for future work given its likely influence on NVP recommendation to patients among clinicians. Interestingly, results from this review only yielded articles published between 2018 and 2022 and predominantly from the USA (none identified from Australia, Canada or New Zealand). This further highlights the lack of research in this space and points to the need for sufficient evidence to enable discussion between health practitioners and people diagnosed with cancer.

### Conclusion

People with a current or previous cancer diagnosis commonly perceive e-cigarettes to be less harmful and safer than conventional cigarettes, while clinicians and healthcare providers remain uncertain about the safety of these devices, both of which have implications for clinical practice. Communication between clinicians and patients regarding use of these devices remains inconsistent due to differences in harm perception and confidence in use. Future study is needed to identify the relationship between decreased harm perception and initiation and use of e-cigarettes among people diagnosed with cancer. Further evidence is also needed on the efficacy of e-cigarettes as a smoking cessation tool for people diagnosed with cancer, given its popularity within this population. As it stands, e-cigarette use is associated with adverse lung and cardiovascular outcomes; however, little is known regarding its potential risk of on cancer treatment efficacy and outcomes. Development of resources for healthcare professionals is also required to aid in discussions and recommendations regarding e-cigarettes between clinicians, people diagnosed with cancer, survivors and supports.

## Data Availability

This study is a scoping review that uses retrospective data already published and publicly available.
